# Transcriptional Profiling of Midguts Prepared from *Trypanosoma/T. congolense*-Positive *Glossina palpalis palpalis* Collected from Two Distinct Cameroonian Foci: Coordinated Signatures of the Midguts’ Remodeling As *T. congolense*-Supportive Niches

**DOI:** 10.3389/fimmu.2017.00876

**Published:** 2017-07-28

**Authors:** Jean M. Tsagmo Ngoune, Flobert Njiokou, Béatrice Loriod, Ginette Kame-Ngasse, Nicolas Fernandez-Nunez, Claire Rioualen, Jacques van Helden, Anne Geiger

**Affiliations:** ^1^Faculty of Science, University of Yaoundé I, Yaoundé, Cameroon; ^2^UMR 177, IRD-CIRAD, CIRAD TA A-17/G, Campus International de Baillarguet, Montpellier, France; ^3^Aix-Marseille University, INSERM, TAGC, Technological Advances for Genomics and Clinics, UMR S 1090, Marseille, France

**Keywords:** field tsetse fly, Cameroonian foci, RNAseq, vector control, trypanosomiasis

## Abstract

Our previous transcriptomic analysis of *Glossina palpalis gambiensis* experimentally infected or not with *Trypanosoma brucei gambiense* aimed to detect differentially expressed genes (DEGs) associated with infection. Specifically, we selected candidate genes governing tsetse fly vector competence that could be used in the context of an anti-vector strategy, to control human and/or animal trypanosomiasis. The present study aimed to verify whether gene expression in field tsetse flies (*G. p. palpalis*) is modified in response to natural infection by trypanosomes (*T. congolense*), as reported when insectary-raised flies (*G. p. gambiensis*) are experimentally infected with *T. b. gambiense*. This was achieved using the RNA-seq approach, which identified 524 DEGs in infected vs. non-infected tsetse flies, including 285 downregulated genes and 239 upregulated genes (identified using DESeq2). Several of these genes were highly differentially expressed, with log2 fold change values in the vicinity of either +40 or −40. Downregulated genes were primarily involved in transcription/translation processes, whereas encoded upregulated genes governed amino acid and nucleotide biosynthesis pathways. The BioCyc metabolic pathways associated with infection also revealed that downregulated genes were mainly involved in fly immunity processes. Importantly, our study demonstrates that data on the molecular cross-talk between the host and the parasite (as well as the always present fly microbiome) recorded from an experimental biological model has a counterpart in field flies, which in turn validates the use of experimental host/parasite couples.

## Introduction

Human African trypanosomiasis [HAT or sleeping sickness; (1)] and animal African trypanosomiasis [AAT or nagana; (2)] are two vector-borne diseases that inflict heavy social and economic burdens on sub-Saharan African populations. Although the number of newly diagnosed HAT cases is decreasing (<10,000 per year) (3), more than 60 million people living in endemic areas are at risk of infection (4). In addition, AAT causes a large amount of livestock loss, which has been estimated as high as US$ 4.5 billion per year (5). HAT is due to either *Trypanosoma brucei gambiense* (Tbg; the chronic form of the disease in West and Central Africa) or *T. b. rhodesiense* (the acute form of the disease in East Africa), which are, respectively, transmitted by *Glossina palpalis* and *G. morsitans*. In contrast, AAT is caused by *T. b. brucei, T. congolense* (Tc; the forest or savannah type), or *T. vivax*, and is transmitted by *G. palpalis* or *G. morsitans*.

Despite differences between Tc and Tbg [reviewed in Ref. (6)], the parasites share several important characteristics. In particular, they are digenetic, meaning that they need to successively infect two different hosts to achieve their life cycle. One of these hosts, a *Glossina* fly, is the vector, whereas the other host is a vertebrate, typically a mammal. These parasites must accomplish a crucial part of their life cycle within their vector, namely their multiplication and maturation into the infectious form that can be transmitted to the vertebrate host while the tsetse fly ingests its blood meal. More specifically, Tc and Tbg undergo sequential differentiations after their ingestion by the fly, from the ingested blood stream form to the vertebrate-infective metacyclic form. The latter differentiation occurs either in the proboscis (for Tc) or in the salivary glands (for Tbg) (7), which is the basis for their respective classification into two different subgenera, *Nannomonas* and *Trypanozoon* (8). They also share the ability to excrete/secrete a number of proteins, some of which are considered to be involved in their establishment in the tsetse midgut and/or in the pathogenic process developed within the vertebrate host (9–12). Finally, the establishment of both Tc and Tbg in the *G. palpalis* vector is reported to be favored by *Sodalis glossinidius*, the secondary symbiont inhabitant of the tsetse gut (13). This finding demonstrates the occurrence in naturally infected field tsetse flies of a tripartite interaction (fly/trypanosome/gut bacteria) already reported to occur in experimentally infected insectary flies (14–18).

Another similarity between the parasites is that their mantle, which consists of a variant surface glycoprotein, allows them to evade the host’s immune system by means of antigenic variations (19–21), thus rendering ineffective any vaccine approaches to fight HAT or AAT. Nevertheless, progress has been made in rapid diagnosis (22) and therapy that uses a nifurtimox–elfornitine combination in the treatment of the second phase of HAT (23). Furthermore, besides the use of trypanocidal drugs, the incidence of AAT can be lowered by introducing trypanotolerant cattle into AAT-infected area or through the antibody-mediated inhibition of trypanosome-secreted proteins involved in the parasite pathogenic process (24, 25).

Another approach to fight HAT or AAT is by vector control. Diverse strategies are available, including the application of pesticides, the use of sterile males, and the development of paratransgenic approaches (26–32).

The normal status of tsetse flies is considered to be refractory to trypanosome infection, given that artificial or natural infection rates are always low (28, 33–36). Recently, a global transcriptomic analysis was performed (15–17) in the context of an anti-vector strategy, aimed at deciphering the molecular cross-talk occurring between the different participants involved in tsetse infection: the fly, the trypanosome, and the fly gut bacteria, especially the primary (*Wigglesworthia glossinidia*) and secondary (*S. glossinidius*) symbionts. The authors also focused on identifying differentially expressed genes (DEGs) associated with fly susceptibility or refractoriness as a result of fly infection by the trypanosome. These investigations were performed on insectary-raised *G. p. gambiensis* (Gpg) flies that were artificially infected (or not) by Tbg. This study raised the question of whether the results recorded under these experimental conditions could be transposed to what actually occurs under natural conditions in HAT and AAT foci.

To address this question we have conducted similar transcriptomic analyses on *G. p. palpalis* (Gpp) flies infected or not with Tc, collected in two HAT foci in southern Cameroon. Our experimental design involved a different host vector/parasite couple (Gpp/Tc) from what was used in the previous insectary-raised approach. However, as shown above and in support of this approach, several notable characteristics are shared between the Gpp/Tc couple and the previously used Gpg/Tbg couple, including life cycle, sequential differentiation within the vector, transmission modalities, host immune response escape, and pathological effects on susceptible vertebrate hosts, among others. Thus, the objectives of this study were to determine whether or not field-collected tsetse flies react to trypanosome infection under natural conditions similar to insectary flies under experimental conditions, and whether or not Tc induces molecular disruptions in Gpp similar to those provoked by Tbg in Gpg. Importantly, our approach provides novel evidence that validates the use of experimental host/parasite couples in the context of investigating anti-vector strategies.

## Materials and Methods

### Sampling Areas

Tsetse flies were sampled in May and June 2015 in two active HAT foci (Campo and Bipindi), located in the Ocean Division of the southern region of Cameroon. The Campo focus (2°20′N, 9°52′E) is located on the Atlantic coast and extends along the Ntem river. The HAT National Control Program that visits Campo once per year diagnosed 61 novel HAT cases between 2001 and 2011. The passive identification of two cases in 2012 (37) indicates that HAT is still present. The Bipindi focus (3°2′N, 10°22′E) has a typical forest bioecological environment, including equatorial forest and farmland along roads and around villages. This focus has been recognized since 1920 (38) and includes several villages. Sleeping sickness is still present, since approximately 83 HAT cases were identified by the National Control Program in this focus between 1998 and 2011 (Ebo’o Eyenga, personal communication). In addition to HAT cases that involve *G. palpalis* and Tbg, regular global surveys have identified the presence of several other *Glossina* (including Gpp) and *Trypanosoma* species (including Tc) in both foci. Surveys have also identified a variety of domestic and wild animals that serve as reservoirs for diverse *Trypanosoma* species (39–42). As described below, flies were trapped in these areas in order to select non-infected and Tc-infected individuals.

### Fly Sampling, Dissection, and Subsequent RNA Preservation

The May 2015 tsetse fly trapping campaign was conducted in three Campo villages (Ipono, Mabiogo, and Campo-Beach), and the June 2015 campaign was conducted in three Bipindi villages (Lambi, Bidjouka, and Ebiminbang). The geographical positions of the sampling sites were determined by GPS. Tsetse flies were captured using pyramidal traps (43) placed in suitable tsetse fly biotopes. Each trap was installed for four consecutive days, and the flies were collected twice per day.

Prior to handling samples, work stations and dissecting instruments were cleaned with RNase away (Ambion) in order to eliminate any RNases that could degrade sample RNA. Furthermore, tsetse flies were dissected alive to prevent RNA degradation by normal *post mortem* degradation processes. The first step in sample processing consisted in identifying the collected tsetse flies to the species level on the basis of morphological criteria and adapted taxonomic keys (44). Next, the samples were separated into two groups of teneral and non-teneral flies. The non-teneral Gpp flies were dissected in a drop of sterile 0.9% saline solution, according to the midgut dissection protocol developed by Penchenier and Itard (45). The organs were immediately transferred to tubes containing RNAlater (Ambion) for DNA and RNA extraction. These samples were then used for parasite identification by specific PCR amplification, and ultimately for transcriptomic analysis. All tools were carefully cleaned after the dissection of each fly to prevent cross-contamination. During field manipulations, the tubes containing the organs were stored at −20°C for 5 days; subsequently, they were stored in the laboratory at −80°C until use.

### DNA and RNA Extraction

To prepare for extraction, samples stored at −80°C were thawed and RNAlater was removed. The midguts were treated with the NucleoSpin TriPrep extraction kit (Macherey-Nagel) according to the manufacturer’s instructions, which allow the separate extraction of DNA and RNA. RNase free water (40 µl) was added to elute the RNA, and 100 µl of DNA elute solution was added to recover the DNA. RNA quality and the absence of any DNA contamination were checked on an Agilent RNA 6000 Bioanalyzer and quantified using the Agilent RNA 6000 Nano kit (Agilent Technologies, France).

### PCR Amplification

To identify which trypanosome species had infected the sampled tsetse flies, the isolated DNA samples stored at −80°C were thawed and used as a template for PCR amplification with specific primers (Table 1). PCR amplification of parasites was performed as described by Herder et al. (46) and consisted of a denaturing step at 94°C (5 min) followed by 44 amplification cycles, each comprising a denaturing step at 94°C (30 s), annealing at 55°C (30 s), and an extension step at 72°C (1 min). A final extension was performed at 72°C for 10 min. The amplified products were separated on a 2% agarose gel containing ethidium bromide and visualized under UV illumination. Positive (2 ng of reference DNA) and negative controls were included in each PCR amplification experiment. PCR amplifications that gave a positive result were repeated once for confirmation.

**Table 1:** Primers used for PCR amplification of trypanosomes.

Species	Primer sequence	Amplified product (bp)	Reference
*T. brucei* s.l.	5′-CGAATGAATATTAAACAATGCGCAG-3′	164	Masiga et al. (47)
	5′-AGAACCATTTATTAGCTTTGTTGC-3′		
*T. congolense* (“forest” type)	5′-CGAATGAATATTAAACAATGCGCAG-3′	350	Masiga et al. (47)
	5′-AGAACCATTTATTAGCTTTGTTGC-3′		
*T. congolense* (“savannah” type)	5′-CGAATGAATATTAAACAATGCGCAG-3′	341	Moser et al. (48)
	5′-AGAACCATTTATTAGCTTTGTTGC-3′		

*T, Trypanosoma; s.l., sensu lato*.

### RNA-Seq Processing

#### Preparation of cDNA Libraries

Total RNA from 10 Gpp flies (5 non-infected flies and 5 flies infected by Tc s.l.) was assayed using the TruSeq mRNA-seq Stranded v2 Kit (Illumina), according to the manufacturer’s instructions. Briefly, 4 µg of total RNA were used for poly(A)-selection to generate 120–210 bp cDNA fragments (mean size: 155 bp) after an 8-min elution-fragmentation incubation. Each library was barcoded using TruSeq Single Index (Illumina), according to the manufacturer’s instructions. After library preparation, Agencourt AMPure XP (Beckman Coulter, Inc.) was used to select 200- to 400-bp size libraries. Each library size distribution was examined using the Bioanalyzer with a High Sensitivity DNA chip (Agilent) to ensure that the samples had the proper size and that they were devoid of any adaptor contamination. The sample concentration was quantified on Qubit with the Qubit^®^ dsDNA HS Assay Kit (Life Technologies). Each library was then diluted to 4 nM and pooled at an equimolar ratio.

#### NextSeq-500 Sequencing

For sequencing, 5 µL of pooled libraries (4 nM) were denatured with 5 µl NaOH (0.2 N) according to the manufacturer’s instructions. Following a 5-min incubation, 5 µl of Tris–HCl (200 mM; pH 7) were added, and 20 pM of the pooled libraries were diluted with HT1 to a 1.6-pM final concentration. As a sequencing control, a PhiX library was denatured and diluted according to the manufacturer’s instructions, and 1.2 µl were added to the sample of denatured and diluted pooled libraries before loading. Finally, the libraries were sequenced on a high-output flow cell (400M clusters) using the NextSeq^®^ 500/550 High Output Kit v2 (150 cycles; Illumina) in paired-end 75/75 nt mode, according to the manufacturer’s instructions.

Datasets for the reads are available from the NCBI, GEO submission, accession number GSE98989.

### Bioinformatics Analysis

#### Workflow

The successive tasks of the bioinformatics analysis were managed using a Snakemake workflow (49). This workflow enables the reproduction of all analyses from the raw read files and is available from the supporting Web site.^1^

#### Reference Genomes

The *G. palpalis* genomic sequences (Glossina-palpalis-IAEA_SCAFFOLDS_GpapI1.fa) and annotations (Glossina-palpalis-IAEA_BASEFEATURES_GpapI1.1.gff3) were downloaded from VectorBase (50). For the annotation of Tc genes, the reference genome (TriTrypDB-9.0_TcongolenseIL3000.gff) was downloaded from TriTrypDB (51), whereas the *Drosophila melanogaster* reference genome (Drosophila_melanogaster.BDGP6.30.gff3) was downloaded from Flybase (52).

#### Lane Merging

Since each sample was sequenced on four lanes, the original fastq-formatted read files were merged to produce two files per sample (one for each paired-end extremity).

#### Read Quality Control

FastQC^2^ was run on the raw reads in order to check their quality.

#### Read Mapping

Raw reads were mapped onto the genome with the local alignment algorithm Subread-align (53) in paired-ends mode with at most 10 mismatches. Read mapping statistics were computed using samtools flagstats (54) and are summarized in Table S1 in Supplementary Material.

#### Read Counts per Gene

The number of read pairs (fragments) per gene was counted using the featureCounts tool from the Subread package (55), including the option “feature type” to only count reads overlapping transcripts.

#### Visualization

Genome maps were generated using the Integrative Genomic Viewer (56).

#### Detection of DEGs

Differential gene expression analysis was performed using the SARTools R package (57), which separately runs DESeq2 (58) and egdeR (59) as well as generates readable reports.

#### Identification of Orthologs between Gpp and *D. melanogaster*

Because the *G. palpalis* genome is inadequately assembled and annotated, our functional interpretation of the DEGs relied on a comparative genomics approach. This was based on the identification of bidirectional best hits (BBH) between all sequences of *G. palpalis* and *D. melanogaster* (assembly BDGP6). BBH were identified using blastp (60) and the BLOSUM45 substitution matrix, and by setting a threshold of 10^−5^ on the expected score.

#### Functional Enrichment of DEGs

Identification of functions associated with the DEGs was based on *Drosophila* orthologs of the DEGs (ortho-DEG). Functional enrichment was separately performed using the DAVID (61) and g:Profiler (62) tools. The Bonferroni correction was used to obtain the enrichments of these functions, with a threshold set at 10^−3^.

#### Pathway Mapping of DEGs

*Drosophila* orthologs of the DEGs were loaded into the metabolic cellular overview of BioCyc (63) in order to highlight the pathways affected by the infection.

#### Statistical Treatment of Entomological Data

Entomological data, as well as all other calculations, were evaluated using the statistical package SPSS version 2.0. Spreadsheets were made using Microsoft Office Excel 2007.

## Results

### Entomological Data

A total of 1,991 tsetse flies were collected during the entomological survey (775 flies from Campo and 1,216 flies from Bipindi). The Campo fly population was composed of Gpp (95.61%), *Glossina caliginea* (2.06%), *Glossina palicera* (1.87%), and *Glossina nigrofusca* (0.52%). Two tsetse fly species were identified at the Bipindi focus: Gpp (99.34%) and *G. palicera* (0.66%). The mean apparent density was 4.24 flies per trap per day; however, this parameter was highly variable between the different villages and was higher in Bipindi (8.1) than in Campo (3.52) (Table 2). The frequency of teneral flies was typically low in both Bipindi (0.08%) and Campo (1.16%). These data are roughly in line with data recorded in 2007/2008 (13), although the rate of teneral flies was much lower in the present study. Only 1,245 of the trapped 1,991 tsetse flies were dissected, since 10 flies were teneral and 736 flies were desiccated.

**Table 2:** Entomological field data from the Bipindi and Campo foci.

Focus	Village	Number of traps	Number of tsetse flies captured	ADT	Number of teneral tsetse flies (%)	Number of tsetse flies dissected
Campo	Ipono	15	161	2.68	4 (2.48)	110
	Beach	15	341	4.55	3 (0.87)	264
	Mabiogo	18	273	3.21	2 (0.73)	228
	Total Campo	48	775	3.52	9 (1.16)	602
Bipindi	Bidjouka	23	608	5.28	1 (0.16)	278
	Lambi	12	486	8.1	0 (0)	303
	Ebimimbang	15	122	1.74	0 (0)	72
	Total Bipindi	50	1,216	4.96	1 (0.50)	653
Total		98	1,991	4.24	10 (0.50)	1,255

*ADT, apparent density per trap per day*.

### PCR Identification of Trypanosome Species in the Tsetse Midgut

The number of flies carrying single or mixed trypanosome infections is presented in Table 3. Of the 337 Campo flies analyzed, 25 (7.41%) were infected by the Tc “forest” type, 16 (4.74%) by the Tc “savanah” type, and 14 (4.15%) by both parasites. In contrast, Bipindi flies only carried the Tc “forest” type (8.33%). Table S1 in Supplementary Material details the characteristics of the different samples including those used for transcriptomic analyses.

**Table 3:** Number of *Trypanosoma congolense* s.l. simple and mixed infections by village.

Focus	Village	Number of tsetse flies analyzed	Number of flies infected with TcF	Number of flies infected with TcS	Number of flies carrying a mixed infection
Campo	Ipono	63	2	1	1
	Beach	170	15	8	7
	Mabiogo	104	8	7	6
	Total Campo	337	25	16	14
Bipindi	Bidjouka	40	1	0	0
	Lambi	33	5	0	0
	Ebimimbang	11	1	0	0
	Total Bipindi	84	7	0	0
Total		421	32	16	14

*TcF, T. congolense “forest” type; TcS, T. congolense “savannah” type*.

### Raw Data

**Figure 1:**
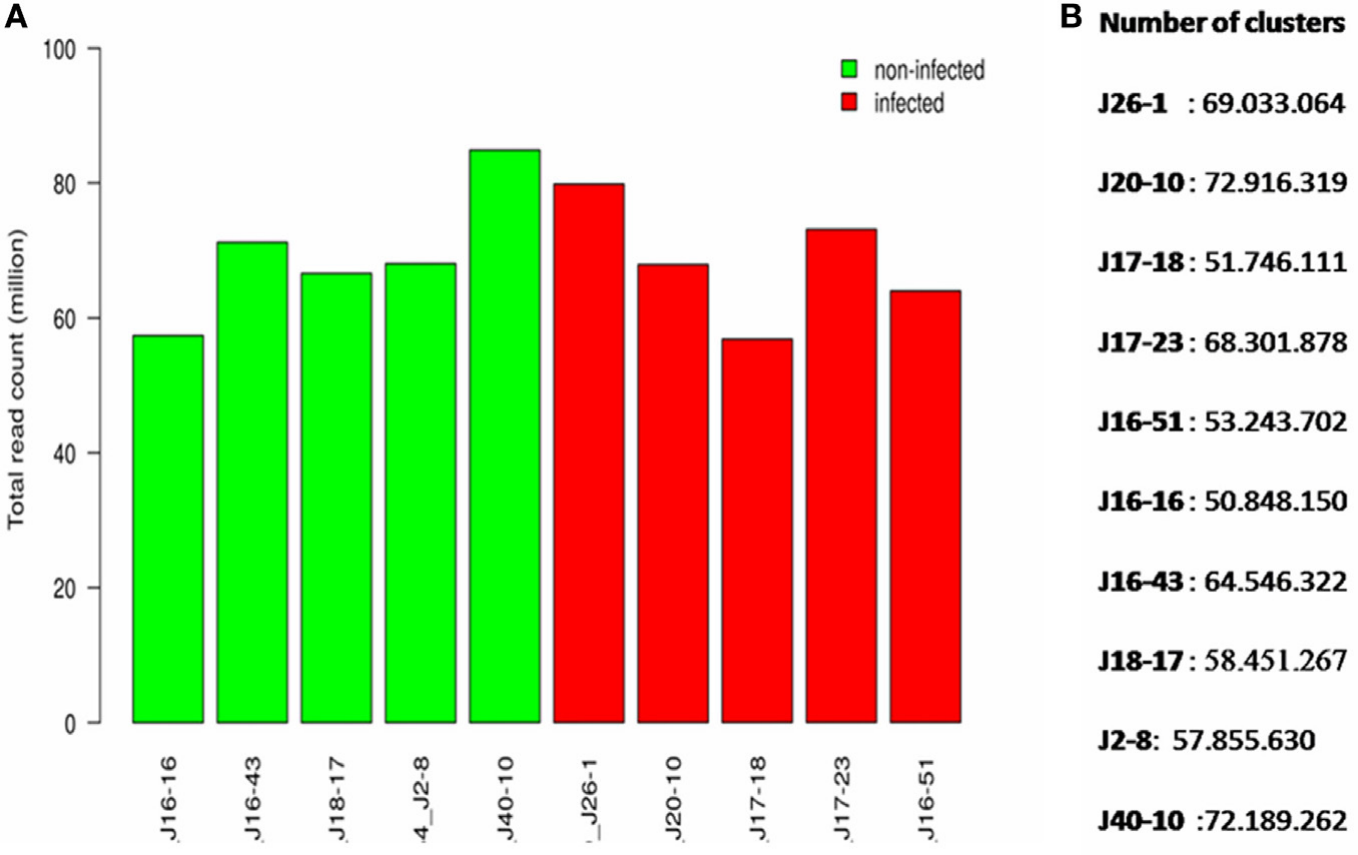
The score quality of different sequences of clusters, presented base by base. Green indicates a good quality. **(A)** Total read count per sample (millions). **(B)** Number of clusters per sample.

The sequencing of libraries produced a total of 400 million reads (theoretically 40 million reads per sample), which represent a satisfactory sequencing depth for subsequent differential gene expression analysis (Figure 1). Out of the 328 raw clusters generated, 77.6% were successfully filtered, with each sample producing 50–72 million clusters (mean: 61 million clusters) (Figure 1). Sequencing also revealed a total of 31,320 contigs distributed in 3,926 scaffolds with a mean size of 96,817 bp (varying in size from 545 bp to 3.6 Mb).

### Mapping on Gpp

RNA-seq sequencing produced an average of 124 million reads per sample. From this, 111.45 million reads (83.6%) were mapped onto the genome of Gpp, 103.68 M (73.7%) of which were properly paired (Table 4).

**Table 4:** Read mapping statistics.

Mapping on	Mapped reads (%)	Properly paired reads (%)	Singleton reads (%)	QC-passed reads
*Glossina*	111.45 M (89.87)	103.68 M (83.61)	3.62 M (2.92)	123.8 M
*Trypanosoma*	0.3 M (0.24)	0.13 M (0.11)	0.15 M (0.12)	123.8 M

*M, millions of reads*.

### Mapping on Tc

Reads were also mapped onto the trypanosome genome in order to validate the infection status of the samples, as well as to investigate the role played by the trypanosome in this molecular dialog. Since trypanosome cells represented a small fraction of the analyzed material, only a smaller fraction of the reads could be mapped. Specifically, 300,302.5 (0.24%) reads were mapped from an average of 124 million reads per sample. This resulted in 137,305.4 (0.11%) properly paired reads and 157,712.5 (0.12%) singletons (Table 4).

### DEGs between Infected and Non-Infected Flies

**Figure 2:**
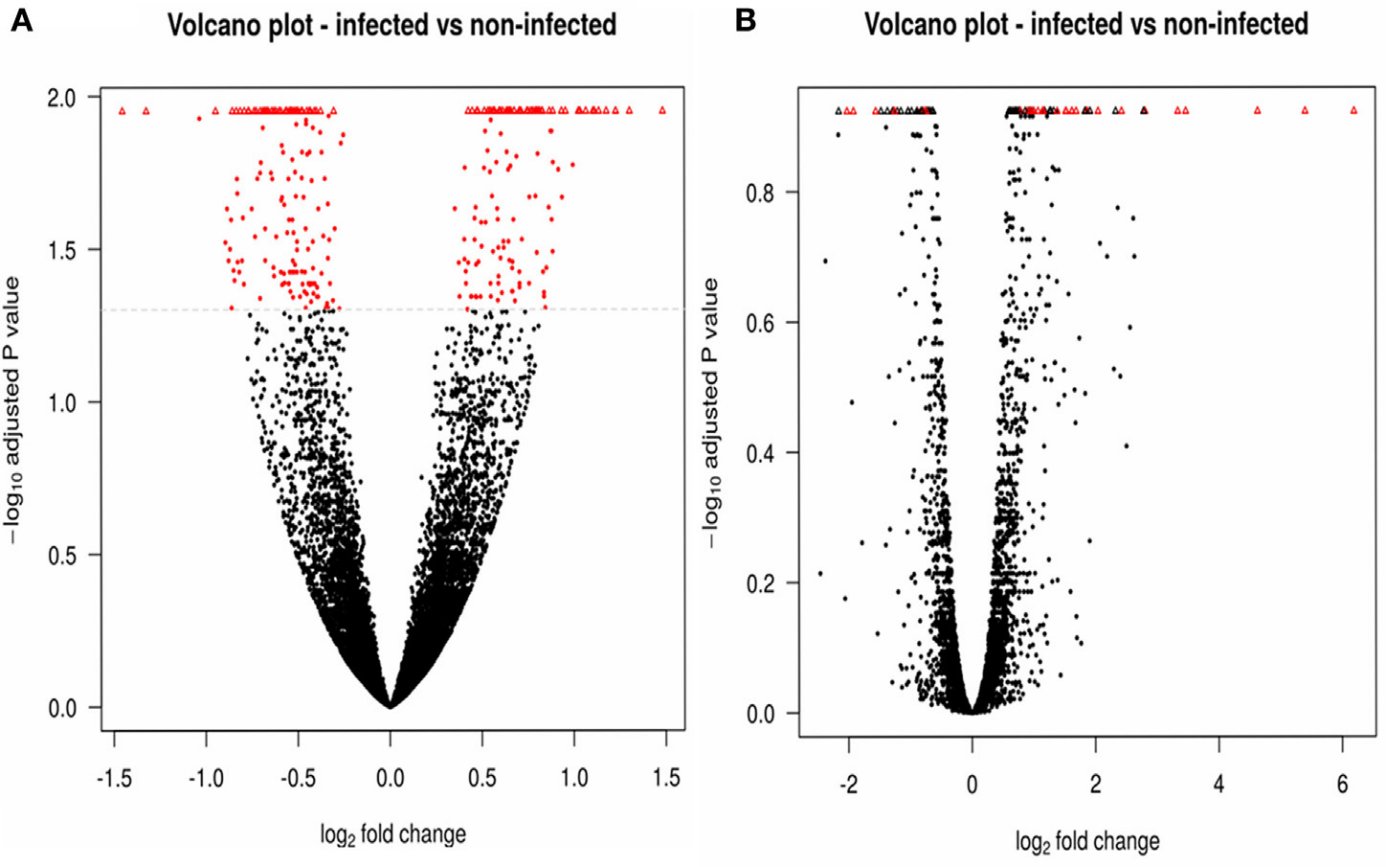
Comparisons of significantly differentially expressed genes. DESeq2 **(A)** and edgeR **(B)** results are illustrated by volcano plots, in which the differentially expressed features are shown in red. Upregulated genes are thus observed as positive values, and downregulated genes as negative values. Triangles correspond to features where the log of the adjusted *p*-value is too low or too high to be displayed on the plot.

We used DESeq2 (Table 5) to detect genes that were differentially expressed between the five infected and five non-infected samples. When a Benjamini–Hochberg corrected *p*-value lower than 0.05 was applied, 524 genes were observed to be significantly differentially expressed in infected vs. non-infected flies, among which 285 genes were downregulated and 239 were upregulated. A similar DEG analysis was performed using edgeR (Table 5), which identified only 20 downregulated genes and 53 upregulated genes. Figure 2 presents the volcano plots produced by DESeq2 (Figure 2A) and edgeR (Figure 2B); genes that were significantly (*p*-value <0.05) differentially expressed and with a fold change of log2 (fold change) >2 (i.e., upregulated genes) or log2 (fold change) <−2 (i.e., downregulated genes) were considered relevant.

**Table 5:** Differentially expressed genes.

	Downregulated	Upregulated	Total
DESeq2	285	239	524
edgeR	20	53	73
Common	20	46	66
Total	285	246	531

### Functional Annotation

To understand the roles of DEGs associated with tsetse fly infection by Tc, identifiers of the *Drosophila* orthologs of the *Glossina* DEGs were examined using the DAVID-functional enrichment tool. Separately, we analyzed the 290 downregulated and 213 upregulated genes reported by DESeq2 and identified 207 *Drosophila* best hits (121 downregulated and 86 upregulated genes). The same analysis by edgeR only resulted in 25 DEG orthologs (6 downregulated and 19 upregulated genes). This reduced number of orthologs could possibly be due to the incomplete assembly and annotation of the *Glossina* genome and/or the high stringency of the BBH criterion (which discards the case where several tsetse fly proteins have the same closest hit in *Drosophila*). These DEGs were examined using DAVID, which compares the list of input genes with a variety of functional annotations. This analysis was focused on the three primary categories of the Gene Ontology annotation: biological process (BP), molecular function (MF), and cellular component (CC). The list of different features is provided in Table 6.

**Table 6:** Functional annotation of differentially expressed genes (DEGs).

Category	Term	Number of DEG	*p*-value	Log2 (fold enrichment)
**DEGs identified with DESeq2**				
GOTERM_BP_FAT	Amine biosynthetic process	6	2.80E−05	15.70
SP_PIR_KEYWORDS	Amino acid biosynthesis	3	2.00E−03	42.80
GOTERM_MF_FAT	ATPase activity, uncoupled	5	9.10E−02	−2.90
GOTERM_MF_FAT	ATP-dependent helicase activity	4	5.80E−02	−4.50
GOTERM_MF_FAT	ATP-dependent RNA helicase activity	4	1.10E−02	−8.40
GOTERM_BP_FAT	Axon guidance	6	2.90E−03	5.90
GOTERM_BP_FAT	Axonal defasciculation	2	8.70E−02	21.50
GOTERM_BP_FAT	Axonogenesis	6	1.20E−02	4.20
GOTERM_BP_FAT	Carboxylic acid biosynthetic process	5	9.90E−04	10.80
GOTERM_MF_FAT	Cation binding	18	6.10E−02	1.50
GOTERM_BP_FAT	Cell morphogenesis	7	8.10E−02	2.20
GOTERM_BP_FAT	Cell morphogenesis involved in differentiation	6	5.70E−02	2.80
GOTERM_BP_FAT	Cell morphogenesis involved in neuron differentiation	6	5.00E−02	2.90
GOTERM_BP_FAT	Cell motion	6	5.20E−02	2.90
GOTERM_BP_FAT	Cell part morphogenesis	6	7.60E−02	2.60
GOTERM_BP_FAT	Cell projection morphogenesis	6	7.20E−02	2.60
GOTERM_BP_FAT	Cell recognition	3	4.40E−02	8.70
GOTERM_BP_FAT	Cellular amino acid biosynthetic process	5	5.40E−05	22.40
GOTERM_BP_FAT	Chemical homeostasis	3	7.70E−02	6.30
GOTERM_MF_FAT	Coenzyme binding	4	6.40E−02	4.30
GOTERM_CC_FAT	Cytosol	6	9.30E−02	−2.40
INTERPRO	DEAD-like helicase, N-terminal	4	6.20E−02	−4.30
GOTERM_BP_FAT	Defasciculation of motor neuron axon	2	6.20E−02	30.70
SMART	DEXDc	4	4.30E−02	−4.90
GOTERM_BP_FAT	Di-, tri-valent inorganic cation transport	3	3.40E−02	10.10
INTERPRO	DNA/RNA helicase, C-terminal	4	6.20E−02	−4.30
INTERPRO	DNA/RNA helicase, DEAD/DEAH box type, N-terminal	4	1.60E−02	−7.40
GOTERM_MF_FAT	Electron carrier activity	5	2.50E−02	4.40
GOTERM_MF_FAT	Enzyme inhibitor activity	3	9.90E−02	5.50
GOTERM_CC_FAT	Eukaryotic translation initiation factor 3 complex	4	4.40E−04	−24.50
GOTERM_MF_FAT	Glutamate synthase activity	2	2.10E−02	93.60
GOTERM_BP_FAT	Glutamine family amino acid biosynthetic process	2	9.50E−02	19.60
GOTERM_BP_FAT	Glutamine family amino acid metabolic process	3	1.50E−02	15.40
GOTERM_BP_FAT	Glutamine metabolic process	2	6.20E−02	30.70
SP_PIR_KEYWORDS	Helicase	4	6.80E−02	−4.20
INTERPRO	Helicase, superfamily 1 and 2, ATP-binding	4	6.00E−02	−4.40
SMART	HELICc	4	4.30E−02	−4.90
SP_PIR_KEYWORDS	Heme	3	8.40E−02	6.10
GOTERM_MF_FAT	Heme binding	4	2.90E−02	5.90
GOTERM_BP_FAT	Homeostatic process	4	8.90E−02	3.70
SP_PIR_KEYWORDS	Hydrolase	14	6.20E−02	1.70
SP_PIR_KEYWORDS	Initiation factor	3	3.40E−02	−10.10
GOTERM_CC_FAT	Intracellular non-membrane-bounded organelle	17	3.60E−03	−2.00
GOTERM_CC_FAT	Intracellular organelle lumen	12	1.00E−02	−2.30
GOTERM_MF_FAT	Ion binding	18	6.40E−02	1.50
GOTERM_MF_FAT	Iron ion binding	6	1.10E−02	4.30
KEGG_PATHWAY	Limonene and pinene degradation	3	6.60E−02	6.60
GOTERM_MF_FAT	Lipase activity	3	9.30E−02	5.70
SP_PIR_KEYWORDS	Lipid-binding	2	4.80E−02	39.90
GOTERM_CC_FAT	Membrane-enclosed lumen	12	1.20E−02	−2.20
GOTERM_MF_FAT	Metal ion binding	18	5.10E−02	1.50
INTERPRO	Mitochondrial substrate carrier	3	3.10E−02	10.60
INTERPRO	Mitochondrial substrate/solute carrier	3	3.30E−02	10.20
GOTERM_CC_FAT	Mitochondrion	8	6.00E−02	2.10
GOTERM_BP_FAT	Mitotic spindle elongation	4	6.50E−02	−4.20
GOTERM_MF_FAT	MRNA binding	6	3.70E−02	−3.20
GOTERM_BP_FAT	ncRNA metabolic process	12	3.10E−07	−7.50
GOTERM_BP_FAT	ncRNA processing	12	6.00E−09	−10.80
GOTERM_BP_FAT	Neuron development	6	9.30E−02	2.40
GOTERM_BP_FAT	Neuron projection development	6	5.00E−02	2.90
GOTERM_BP_FAT	Neuron projection morphogenesis	6	4.90E−02	2.90
GOTERM_BP_FAT	Neuron recognition	3	4.40E−02	8.70
GOTERM_BP_FAT	Nitrogen compound biosynthetic process	6	1.10E−02	4.20
GOTERM_CC_FAT	Non-membrane-bounded organelle	17	3.60E−03	−2.00
GOTERM_CC_FAT	Nuclear lumen	12	4.10E−04	−3.30
GOTERM_CC_FAT	Nucleolus	11	2.20E−09	−13.50
SP_PIR_KEYWORDS	Nucleus	18	1.90E−02	−1.80
GOTERM_CC_FAT	Organelle lumen	12	1.00E−02	−2.30
GOTERM_BP_FAT	Organic acid biosynthetic process	5	9.90E−04	10.80
GOTERM_BP_FAT	Oxidation reduction	8	3.10E−02	2.50
GOTERM_MF_FAT	Phospholipase activity	3	3.60E−02	9.70
SP_PIR_KEYWORDS	Phosphoprotein	16	4.90E−02	−1.70
GOTERM_BP_FAT	Positive regulation of protein kinase cascade	2	8.50E−02	−22.30
GOTERM_CC_FAT	Preribosome	3	3.40E−03	−31.50
SP_PIR_KEYWORDS	Protein biosynthesis	5	1.70E−02	−5.00
GOTERM_BP_FAT	Pseudouridine synthesis	2	9.90E−02	−19.10
GOTERM_MF_FAT	Purine NTP-dependent helicase activity	4	5.80E−02	−4.50
GOTERM_BP_FAT	Regulation of translational initiation	3	1.10E−02	−18.20
SP_PIR_KEYWORDS	Ribonucleoprotein	6	1.30E−02	−4.20
GOTERM_CC_FAT	Ribonucleoprotein complex	13	6.50E−05	−3.70
GOTERM_BP_FAT	Ribonucleoprotein complex biogenesis	12	2.80E−09	−11.60
GOTERM_BP_FAT	Ribosome biogenesis	12	7.10E−11	−16.00
SP_PIR_KEYWORDS	Ribosome biogenesis	5	1.20E−04	−18.60
GOTERM_MF_FAT	RNA binding	14	9.00E−05	−3.60
GOTERM_MF_FAT	RNA helicase activity	4	1.50E−02	−7.50
INTERPRO	RNA helicase, ATP-dependent, DEAD-box, conserved site	3	4.20E−02	−9.00
INTERPRO	RNA helicase, DEAD-box type, Q motif	4	6.50E−03	−10.20
GOTERM_BP_FAT	RNA modification	3	7.10E−02	−6.70
GOTERM_BP_FAT	RNA processing	14	1.40E−05	−4.30
SP_PIR_KEYWORDS	RNA-binding	10	2.20E−05	−6.40
GOTERM_MF_FAT	RNA-dependent ATPase activity	4	1.10E−02	−8.40
GOTERM_BP_FAT	RRNA metabolic process	10	2.10E−10	−22.30
GOTERM_BP_FAT	RRNA modification	2	9.90E−02	−19.10
GOTERM_BP_FAT	RRNA processing	10	2.10E−10	−22.30
SP_PIR_KEYWORDS	RRNA processing	5	1.50E−04	−17.70
COG_ONTOLOGY	Secondary metabolites biosynthesis, transport, and catabolism	3	3.40E−02	8.60
GOTERM_CC_FAT	Small nuclear ribonucleoprotein complex	4	1.80E−02	−6.80
GOTERM_CC_FAT	Small nucleolar ribonucleoprotein complex	4	4.20E−05	−49.00
GOTERM_CC_FAT	Small-subunit processome	3	1.70E−03	−44.10
GOTERM_BP_FAT	Spindle elongation	4	6.70E−02	−4.20
GOTERM_MF_FAT	Tetrapyrrole binding	4	2.90E−02	5.90
GOTERM_BP_FAT	Translation	9	2.10E−02	−2.60
GOTERM_BP_FAT	Translational initiation	4	1.80E−02	−7.00
SP_PIR_KEYWORDS	Transport	7	5.60E−02	2.50
SP_PIR_KEYWORDS	WD repeat	7	1.00E−02	−3.80
SMART	WD40	7	8.30E−03	−3.80
INTERPRO	WD40 repeat	7	1.80E−02	−3.30
INTERPRO	WD40 repeat, conserved site	5	5.30E−02	−3.50
INTERPRO	WD40 repeat, region	6	2.60E−02	−3.60
INTERPRO	WD40 repeat, subgroup	7	7.80E−03	−4.00
INTERPRO	WD40/YVTN repeat-like	8	5.90E−03	−3.60
**DEGs identified with DESeq2**				
GOTERM_BP_FAT	One-carbon metabolic process	2	6.00E−02	−27.2
GOTERM_MF_FAT	Carboxylesterase activity	2	8.40E−02	20.4
GOTERM_MF_FAT	Lipase activity	2	7.90E−02	21.7
GOTERM_MF_FAT	Phospholipase activity	2	4.80E−02	36.6

*Categories: GOTERM_BP_FAT, biological process; GOTERM_CC_FAT, cellular component; GOTERM_MF_FAT, molecular function*.

*SMART & INTERPRO, protein domains; SPIR_KEYWORD, protein information resource provided by SWISSPROT and UniProt*.

*CDG_ONTOLOGY, cluster orthology group*.

*Black fonts: downregulated DEGs; red fonts: upregulated DEGs*.

**Figure 3:**
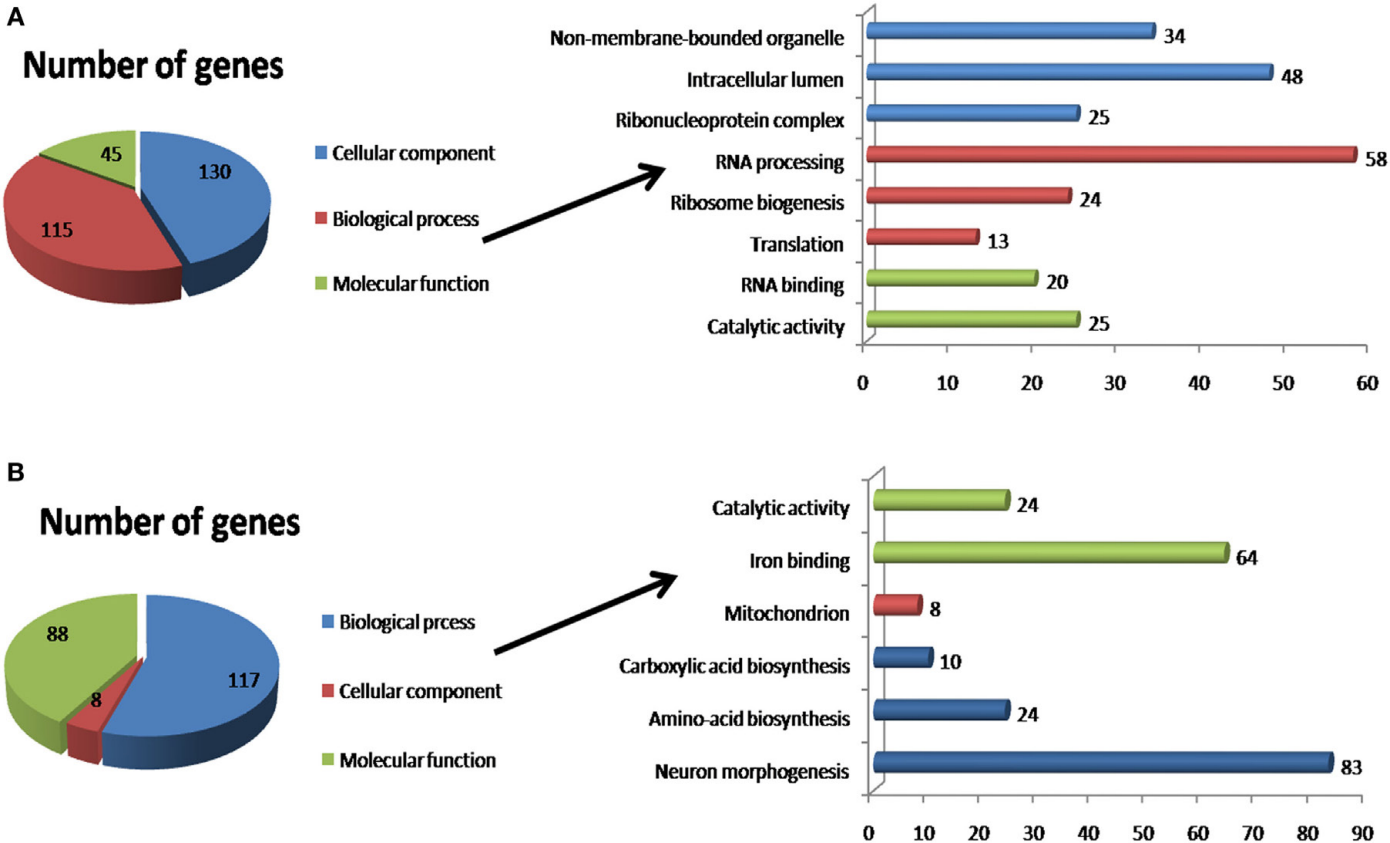
Functional annotation of differentially expressed genes using DAVID. **(A)** Downregulated genes identified by DESeq2. **(B)** Upregulated genes identified by DESeq2. The *x*-axis indicates the number of genes enriched for the term, and the *y*-axis indicates the functional classes that were differentially expressed.

The 285 downregulated genes identified by DESeq2 in flies infected with Tc mainly belonged to the BP category, in which the major functional classes were RNA processing (58 genes; 23.48%), ribosome biogenesis (24 genes; 9.71%), and translation (13 genes; 5.26%); the other genes corresponded to several poorly represented classes. The MF category included the functional RNA binding classes (20 genes; 8.09%) and catalytic activity (25 genes; 10.12%). Finally, the CC category included the intracellular lumen (48 genes; 19.43%), non-membrane-bound organelle (34 genes; 13.75%), and ribonucleoprotein complex (25 genes; 10.12%) functional classes (Figure 3).

In addition, 239 DEGs were overexpressed in Tc-infected tsetse flies. These DEGs encoded proteins corresponding to the same three primary ontology categories. The BP category include the neuron morphogenesis functional class (83 genes; 38.96%), amino acid biosynthesis (24 genes; 11.26%), and carboxylic acid biosynthesis (10 genes; 4.7%). The MF category included the iron binding (64 genes, 30%) and catalytic activity (24 genes; 11.26%) functional classes. Finally, the CC category only included the mitochondrion functional class (8 genes; 3.75%).

As already shown for DEGs, edgeR provides a much lower number of functional annotations than DESeq2. Here, using edgeR, fly genes that displayed an increased expression in response to Tc infection were found to belong to the MF category, with only three functional classes: phospholipase activity (2 genes; 10.5%), lipase activity (2 genes; 10.5%), and carboxylesterase activity (2 genes; 10.5%). Finally, expression was decreased for only two genes belonging to the one-carbon metabolic process term (BP category).

### Functional Enrichment of DEGs

To make our analysis more focused and efficient, a functional enrichment was performed to refine the list of tsetse fly DEGs in which expression was influenced by Tc infection. We therefore combined fold enrichment and the *p*-value at a 5% threshold, which allowed applying a Bonferroni correction to eliminate false positives. The Bonferroni correction threshold was fixed at α = 10^−2^, and all functionality with a Bonferroni value below this threshold was considered to be due to trypanosome infection.

Following this correction, 16 functional classes were found to be selectively altered by trypanosome infection. These classes are mainly involved in the transcription process, including (a) RNA related processes (rRNA processing, rRNA metabolic process, ncRNA processing, ncRNA metabolic processes, and RNA processing), involving 63 DEGs; (b) monitoring processes related to the synthesis of the ribonucleoprotein complex (ribonucleoprotein complex biogenesis and ribonucleoprotein complex), involving 29 DEGs; (c) RNA binding, involving 24 DEGs; (d) nucleolus biogenesis (nucleolus and nuclear lumen), involving 23 DEGs; (e) ribosome synthesis, involving 17 DEGs; and (f) eukaryotic translation factor 3 complex, involving 4 DEGs. In contrast to the transcription process, which involved 160 DEGs, the BPs of organic acid synthesis (amine biosynthetic process, cellular amino acid biosynthetic process, carboxylic acid biosynthetic process, and organic acid biosynthetic process) that were found to be activated by trypanosome infection only involved 21 DEGs (Table 7).

**Table 7:** Bonferroni correction for differentially expressed genes (DEGs) enriched functionalities.

Category	Term	Number of DEGs	Bonferroni
	**Downregulated DESEQ2**		
GOTERM_BP_FAT	Ribosome biogenesis	12	3.90E−08
GOTERM_BP_FAT	rRNA processing	10	5.80E−08
GOTERM_BP_FAT	rRNA metabolic process	10	5.80E−08
GOTERM_BP_FAT	Ribonucleoprotein complex biogenesis	12	5.10E−07
GOTERM_BP_FAT	ncRNA processing	12	8.30E−07
GOTERM_BP_FAT	ncRNA metabolic process	12	3.40E−05
GOTERM_BP_FAT	RNA processing	14	1.20E−03
GOTERM_CC_FAT	Nucleolus	11	2.00E−07
GOTERM_CC_FAT	Small nucleolar ribonucleoprotein complex	4	1.90E−03
GOTERM_CC_FAT	Ribonucleoprotein complex	13	2.00E−03
GOTERM_CC_FAT	Nuclear lumen	12	9.40E−03
GOTERM_CC_FAT	Eukaryotic transl. initiation factor 3 complex	4	8.00E−03
GOTERM_MF_FAT	RNA binding	14	1.40E−02
SP_PIR_KEYWORDS	RNA-binding	10	1.80E−03
SP_PIR_KEYWORDS	Ribosome biogenesis	5	4.70E−03
SP_PIR_KEYWORDS	rRNA processing	5	4.00E−03

	**Upregulated DESEQ2**		

GOTERM_BP_FAT	Amine biosynthetic process	6	8.80E−03
GOTERM_BP_FAT	Cellular amino acid biosynthetic process	5	8.50E−03
GOTERM_BP_FAT	Carboxylic acid biosynthetic process	5	9.90E−02
GOTERM_BP_FAT	Organic acid biosynthetic process	5	9.90E−02

*Categories: GOTERM_BP_FAT, biological process; GOTERM_CC_FAT, cellular component; GOTERM_MF_FAT, molecular function*.

*SMART & INTERPRO, protein domains; SPIR_KEYWORD, protein information resource provided by SWISSPROT and UniProt*.

### Associated Metabolic Pathways

**Figure 4:**
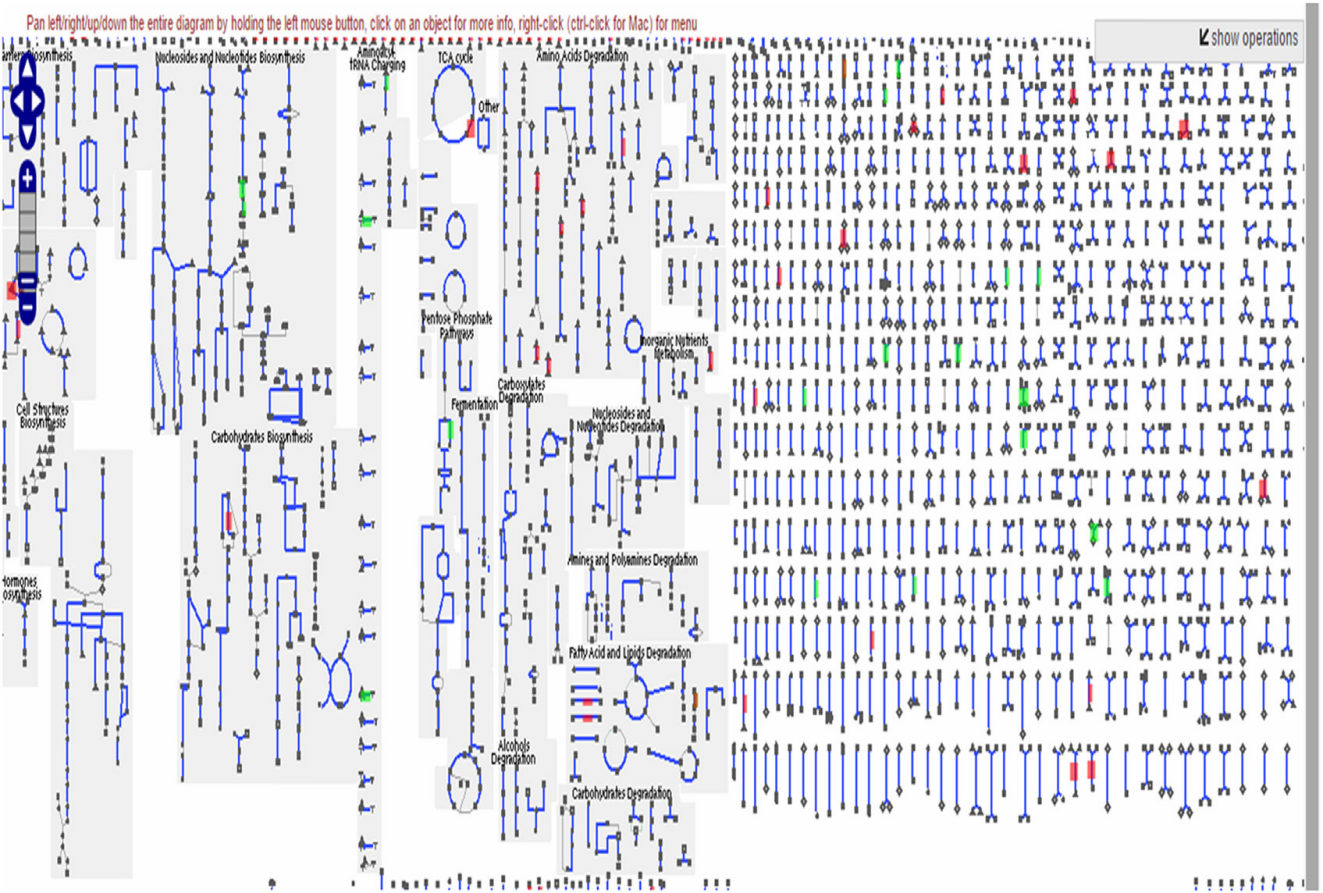
BioCyc metabolic map illustrating the different differentially expressed genes involved in tsetse fly metabolic pathways. The genes activated by the infection are displayed in red, whereas the repressed genes are displayed in green.

The BioCyc metabolic map (Figure 4) and Table 8 illustrate the different pathways that the DEGs are involved in. Among these, the amino acid biosynthesis pathway (which includes the biosynthesis of l-glutamine, l-glutamate, l-serine, l-asparagine, l-aspartate, etc.) is controlled by genes that were shown to be overexpressed following trypanosome infection in the flies. Similarly, DEGs associated with the nucleotide biosynthesis pathway were overexpressed following trypanosome infection, especially uridine monophosphate, an RNA monomer. In contrast, genes involved in the pentose phosphate pathway, namely those implicated in the synthesis of d-ribose 5-phosphate, were downregulated. Phosphorylated pentose is converted by ribose phosphate diphosphokinase into phosphoribosylpyrophosphate, a precursor of nucleotide synthesis. Finally, regarding the carbohydrate biosynthesis pathway, we observed an overexpression of genes encoding malate dehydrogenase, which converts malate into pyruvate.

**Table 8:** Metabolic pathways associated with fly infection.

Pathways	Functions
Amino acid biosynthesis pathway	Synthesis of l-glutamine
	Synthesis of l-glutamate
	Synthesis of l-serine
	Synthesis of l-asparagine
	Synthesis of l-aspartate
	Synthesis of l-valine
	Synthesis of l-proline
	Synthesis of l-isoleucine
Nucleotide biosynthesis pathway	Biosynthesis of uridine monophosphate
Pentose phosphate pathway	Repression of d-ribose-5-phosphate
	Synthesis of orotidine
	Synthesis of pyruvate
Carbohydrate biosynthesis pathway	Biosynthesis of pyruvate
Isolated reactions	Synthesis of tyrosine
	Synthesis of serine
	Synthesis of l-glutamine
	Synthesis of l-glutamate
	Synthesis of l-serine
	Synthesis of l-asparagine
	Synthesis of l-methionine
	Synthesis of l-aspartate
	Synthesis of l-valine
	Synthesis of l-isoleucine
	Synthesis of *N*-acetylglucosamine
	Synthesis of galactosyltransferase
	Synthesis of beta-1,4-manosylglycolipid
	Synthesis of S-adenosyl-l-homocysteine
	Repression of immune cytokines
	Repression of formyltetrahydrofolate DH
Transport	Transport of lipids
	Transport of ATP
	Transport of succinate
	Transport of l-carnitine
	Transport of GTP
	Transport of acid dicarboxylic
	Transport of acid monocarboxylic
	Transport of l-tyrosine
	Transport of l-serine
	Transport of Ca^2+^
	Transport of nucleotide
	Transport of Cyclic GMP
	Transport of proteinogenic amino acid
	Transport of NAD^+^
	Transport of l-fructose
	Transport of GDP
	Transport of fatty acid
	Transporter activity
	Calcium ion binding

Interestingly, a large number of up- and downregulated DEGs were related to a given biosynthetic process meaning that upregulated DEGs encoding amino acids (such as tyrosine, serine, glutamine, and several others) were found in the amino acid synthesis pathway. Other overexpressed genes that were identified are involved in the biosynthesis of galactosyltransferase, *N*-acetylglucosamine, and beta-1,4-manosylglycolipide, which are all molecules that interact with the immune system of the tsetse fly (64–66). In contrast, genes involved in the biosynthesis of cytokines were downregulated in trypanosome-infected flies, as compared to non-infected flies. Similarly, genes involved in folate metabolism (e.g., the biosynthesis of formyltetrahydrofolate dehydrogenase), the main source of energy in flies, were downregulated.

Finally, DEGs involved in the transport of several molecules from the extracellular space toward the cytosol compartment were upregulated. This transport includes molecules with a role in cell nutrition, and nutrients such as lipids, but also ATP, succinate, and l-carnitine (which participates in the degradation of fats).

## Discussion

Understanding the mechanisms involved in tsetse fly susceptibility or refractoriness to trypanosome infection is crucial for developing a novel anti-vector based strategy to control the spread of sleeping sickness and nagana. One recent study was performed within this context to identify genes in Gpg associated with its susceptibility or refractoriness to Tbg infection, using an RNA-seq approach (17). The underlying hypothesis was that some of the genes involved in controlling fly susceptibility/resistance to trypanosome infection could be targeted in order to increase the refractoriness of the fly, thereby decreasing its vector competence while enabling the development of an anti-vector strategy against the disease. As this analysis was performed with insectary flies artificially infected with trypanosomes, it was necessary to verify that similar molecular events occur in field flies naturally infected by trypanosome vs. non-infected flies. To accomplish this, we have chosen the Gpp/Tc couple, whose prevalence (even in HAT foci) is often higher than observed with the Gpp/Tbg couple.

As in the previous study (17), we employed an RNA-seq approach. This provided satisfactory results regarding the mapping of reads on the fly genome, since nearly 75% of the 124 million reads (mean number per sample) were properly paired. However, this was not the case for the Tc genome, which displayed an average of less than 1% of properly mapped reads. This result is not surprising, given that our pre-sequencing manipulations did not target the trypanosome genome. Other contributing factors include the preparation of libraries, which was based on poly(a) selection using Oligo(dT) beads (67) and the *Trypanosoma* genome, which is organized in polycistron units (68).

The infection duration in artificial infection experiments was monitored in the previous report, revealing that the levels of over- or under-expression in DEGs at 3, 10, or 20 days after infection can vary largely (17). In contrast, the present study was performed on tsetse flies sampled in the field, thus neither their age- nor the time-elapsed post-fly infection could be measured. Consequently, the recorded results represent an average level of DEG expression in Gpp flies that may have been infected by trypanosomes (Tc) recently or in the past several days. Similarly, it is possible that non-infected samples could group together flies that were truly never infected with flies that have eliminated their ingested trypanosomes (i.e., “self-cured” or “refractory” flies).

Despite this uncertainty, the results clearly demonstrate a very strong interaction between the parasite and its host/vector, resulting in major transcriptomic changes in the fly. For instance, the level of the “rRNA processing” function in infected vs. non-infected flies was as low as log2 = −22.3. In other words, when the infected flies were captured and dissected, the “rRNA processing” function was 2^22.3^ = 5.16 × 10^6^ fold lower than the value recorded in non-infected flies sampled at the same time and in the same areas. This indicates that the “rRNA processing” function was not effective at that time, and that at least 1 of the 10 DEGs shown to be involved in this function was essentially no longer expressed; however, this does not mean that it could not be reactivated at a later point in a fly’s life.

In this study, we reported that 290 fly genes were downregulated and 213 were upregulated. This type of imbalance is expected to be induced either by a parasite or a symbiont, and to result in disturbing the host metabolism in such a way as to facilitate microorganism establishment (69, 70). In agreement with this, we observed the repression or non-activation of transcription genes that may allow the trypanosome to alter its host’s transcription steps. Furthermore, certain metabolic pathways were downregulated that can prevent the host from synthesizing factors (proteins or metabolites) needed to fight infection (71). In this context and concerning the “Biological Process,” “Cellular Component,” and “Molecular Function” categories, most of the functional classes were associated with the host transcription/translation machinery (translation, RNA binding, ribonucleoprotein complex, ribosome processing helicase, etc.). Only 10% of the DEGs were related to “Catalytic activities.” In contrast, overexpressed DEGs were involved with catalytic activities, cellular activities (morphogenesis, motion, and cell recognition) and, surprisingly, neuron activities (neuron development and neuron recognition). This is coherently illustrated in Table 6, where those “terms” that were over- or under-represented in DEGs (equal to or higher than a fourfold change) and that were identified through functional annotation on the *D. melanogaster* database have been alphabetically classified.

Our identification of the metabolic pathways associated with infection (Table 8) highlights the importance of the amino acid biosynthesis pathway. This provides the parasite with a broad range of amino acids that serve as a valuable source of energy, as previously reported for *T. cruzi*, the parasite causing Chagas disease (72), and microsporidia, a parasite of fishes (73). One such amino acid that we identified is proline, whose synthesis was overexpressed in Gpg infected with Tbg in comparison to non-infected flies (17). We also observed an increase in the biosynthesis of *N*-acetyl-glucosamine, a molecule that can affix itself to lectins that possess a sugar recognition area (74). This process inactivates tsetse fly lectins that are otherwise lethal to procyclic forms of trypanosomes (65), which consequently favor trypanosome installation in the fly vector. Interestingly, this mechanism has also been reported in Gpg infected with Tbg.

As reported in experimental Gpg insectary flies infected with Tbg, we have shown that field-collected Gpp naturally infected with Tc exhibit a strong cytokine repression in comparison to uninfected tsetse flies. This result indicates that strong alteration of the immune system occurred in infected flies, favoring parasite installation. In addition, *Trypanosoma* infection repressed 34 DEGs encoding non-membrane-bound organelles and 48 DEGs encoding expression of the intracellular lumen (an organelle consisting of chromatin). This type of scenario has also been described for the herpes simplex virus type 1, which can modify the structure and dynamics of chromatin through posttranscriptional modification of histone or other chromatin-forming proteins, contributing to their establishment within the host (75).

This is the first study to evaluate the transcriptomic events associated with infection by the Tc trypanosome in field Gpp tsetse flies. Our results establish that field flies naturally infected by trypanosomes display disruptions in their gene expression that result in either overexpression or under-expression of certain fly genes, as similarly observed in experimentally infected insectary flies. Furthermore, molecular disruptions occur in Gpp when infected with Tc, just as in Gpg that have been artificially infected with Tbg. Importantly, these findings indicate that different *Glossina* species infected with different trypanosome species under different conditions display comparable molecular reactions, which validate the use of experimental host/parasite couples for future research programs.

## Author Contributions

AG conceived and designed the experiments. JMTN, FN, BL, GK-N, and NF-N performed the experiments. JMTN, CR, JH, and AG analyzed the data. BL and AG contributed reagents/materials/analysis tools. JMTN, FN, JH, and AG wrote the paper.

## Conflict of Interest Statement

The authors declare that the research was conducted in the absence of any commercial or financial relationships that could be construed as a potential conflict of interest.

## Footnotes

^1^https://github.com/rioualen/gene-regulation.

^2^http://www.bioinformatics.babraham.ac.uk/projects/fastqc/.
