# Supplementary material for: Transcriptional Profiling of Midguts Prepared from Trypanosoma/T. congolense-Positive Glossina palpalis palpalis Collected from Two Distinct Cameroonian Foci: Coordinated Signatures of the Midguts’ Remodeling As T. congolense-Supportive Niches
Source: Front Immunol. 2017 Jul 28;8:876. doi: 10.3389/fimmu.2017.00876 (PMC5532377; doi:10.3389/fimmu.2017.00876)
Supplement: Supplementary file 1 [file table_1.doc]

**Supplementary Table S1.** Read mapping statistics

| Glossina | Mapped reads (%) | Properly paired reads (%) | Singleton reads (%) | QC-passed reads |
| --- | --- | --- | --- | --- |
| J26-1 | 125,058,980 (90.58) | 116,591,168 (84.45) | 4,040,004 (2.93) | 138,066,128 |
| J20-10 | 129,872,362 (89.06) | 119,792,994 (82.14) | 4,710,534 (3.23) | 145,832,638 |
| J17-18 | 90,863,847 (87.80) | 83,749,694 (80.92) | 3,379,607 (3.27) | 103,492,222 |
| J17-23 | 116,641,082 (85.39) | 107,774,428 (78.90) | 4,061,262 (2.97) | 136,603,756 |
| J16-51 | 98,022,521 (92.05) | 92,392,452 (86.76) | 2,723,537 (2.56) | 106,487,404 |
| J16-16 | 92,412,766 (90.87) | 85,939,484 (84.51) | 3,015,912 (2.97) | 101,696,300 |
| J16-43 | 116,643,030 (90.36) | 108,086,960 (83.73) | 3,825,814 (2.96) | 129,092,644 |
| J18-17 | 106,254,728 (90.89) | 98,758,424 (84.48) | 3,456,288 (2.96) | 116,902,534 |
| J2-8 | 105,813,746 (91.45) | 98,890,442 (85.46) | 3,273,354 (2.83) | 115,711,260 |
| J40-10 | 132,974,262 (92.10) | 124,884,702 (86.50) | 3,801,878 (2.63) | 144,378,524 |
| **Mean** | **1,114,557,324 (89.87)** | **1,036,860,748 (83.61)** | **3,628,819 (2.92)** | **123,826,341** |
|  |  |  |  |  |
| Trypanosoma | Mapped reads (%) | Properly paired reads (%) | Singleton reads (%) | QC-passed reads |
| J26-1 | 301,708 (0.22) | 135,810 (0.10) | 160,522 (0.12) | 138,066,128 |
| J20-10 | 280,353 (0.19) | 99,732 (0.07) | 175,517 (0.12) | 145,832,638 |
| J17-18 | 888,372 (0.86) | 720,190 (0.70) | 150,776 (0.15) | 103,492,222 |
| J17-23 | 316,294 (0.23) | 120,492 (0.09) | 189,366 (0.14) | 136,603,756 |
| J16-51 | 167,987 (0.16) | 41,578 (0.04) | 123,777 (0.12) | 106,487,404 |
| J16-16 | 193,502 (0.19) | 43,984 (0.04) | 146,716 (0.14) | 101,696,300 |
| J16-43 | 260,985 (0.20) | 63,124 (0.05) | 194,959 (0.15) | 129,092,644 |
| J18-17 | 195,443 (0.17) | 46,676 (0.04) | 146,541 (0.13) | 116,902,534 |
| J2-8 | 170,859 (0.15) | 43,806 (0.04) | 124,403 (0.11) | 115,711,260 |
| J40-10 | 227,522 (0.16) | 59,662 (0.04) | 164,548 (0.11) | 144,378,524 |
| **Mean** | **300,302.5 (0.24)** | **137,505.4 (0.11)** | **157,712.5 (0.12)** | **123,826,341** |

Yellow underlined: the 5 infected samples under experimentation
